# Swallowing characterization of adult-onset Niemann-Pick, type C1 patients

**DOI:** 10.1186/s13023-024-03241-7

**Published:** 2024-06-11

**Authors:** Beth I. Solomon, Andrea M. Muñoz, Ninet Sinaii, Hibaaq Mohamed, Nicole M. Farhat, Derek Alexander, An Dang Do, Forbes D. Porter

**Affiliations:** 1 Speech-Language Pathology Section, Mark O. Hatfield Clinical Center, National Institutes of Health, Bethesda, MD USA; 2 Eunice Kennedy Shriver National Institute of Child Health and Human Development, National Institutes of Health, Bethesda, MD USA; 3 Biostatistics and Clinical Epidemiology Service, NIH Clinical Center, National Institutes of Health, Bethesda, MD USA

## Abstract

**Background:**

Niemann-Pick disease, type C1 (NPC1) is a rare lysosomal disorder with progressive neurological manifestations, historically recognized as a pediatric disease. However, awareness of the adult-onset (AO) subtype is increasing, often with non-specific symptoms leading to delayed and misdiagnosis. Dysphagia, commonly recognized as a clinical morbidity in NPC1, raises concerns for swallowing safety and aspiration risk. This study aims to characterize swallowing function in AO NPC1, addressing the gap in understanding and clinical management.

**Methods:**

Fourteen AO NPC1 individuals in a prospective natural history study (NCT00344331) underwent comprehensive assessments, including history and physical examinations utilizing the NPC1 severity rating scale, videofluoroscopic swallowing studies with summary interpretive analysis, and cerebrospinal fluid (CSF) collection for biomarker evaluation at baseline visit. Descriptive statistics and multivariate statistical modeling were employed to analyze NPC1 disease covariates, along with the American Speech-Language-Hearing Association National Outcome Measure (ASHA-NOMS) and the NIH Penetration Aspiration Scale (NIH-PAS).

**Results:**

Our cohort, comprised of 14 predominately female (*n* = 11, 78.6%) individuals, had an average age of 43.1 ± 16.7 years at the initial visit. Overall, our AO patients were able to swallow independently with no/minimal cueing, with 6 (43%) avoiding specific food items or requiring more time. Upon risk analysis of aspiration, the cohort demonstrated no obvious aspiration risk or laryngeal aspiration in 8 (57%), minimal risk with intermittent laryngeal penetration and retrograde excursion in 5(36%), and moderate risk (7%) in only one. Dietary modifications were recommended in 7 (50%), particularly for liquid viscosities (*n* = 6, 43%) rather than solids (*n* = 3, 21%). No significant correlations were identified between swallowing outcomes and NPC1-related parameters or CSF biomarkers.

**Conclusion:**

Despite the heterogeneity in NPC1 presentation, the AO cohort displayed functional swallowing abilities with low aspiration risk with some participants still requiring some level of dietary modifications. This study emphasizes the importance of regular swallowing evaluations and management in AO NPC1 to address potential morbidities associated with dysphagia such as aspiration. These findings provide clinical recommendations for the assessment and management of the AO cohort, contributing to improved care for these individuals.

## Introduction

Niemann-Pick disease, type C1 (NPC1) is an ultrarare autosomal recessive lysosomal disorder characterized by loss-of-function alterations in either *NPC1* or *NPC2* genes [1]. While 95% of cases are caused by variants in *NPC1*, impairment in either of these genes leads to dysfunction in intracellular cholesterol trafficking and subsequent accumulation of unesterified cholesterol in late endosomes and lysosomes throughout the body [2]. The estimated incidence of NPC1 is approximately 1 per 100,000 births [1], and the timing of neurological onset is commonly categorized into four groups: infantile (< 6 years), juvenile (6-15 years), adult (> 15 years), and no neurological onset [3–5]. Despite NPC1 historically being regarded as a pediatric disease, subsequent studies have supported the existence of an adult form [5–9]. However, due to the rarity of NPC1 and the gradual onset of non-specific symptoms, adult onset (AO) is frequently underdiagnosed, often misdiagnosed, and continues to remain poorly understood [3, 10]. Several AO cases have been described in the literature that initially presented with psychiatric symptoms years before neurological disease onset, which may further explain the delay in a NPC1 diagnosis seen among some individuals [1, 9, 11, 12]. Prior studies have described AO individuals with onset of neurological symptoms between 18 to 68 years [1, 5, 8, 9, 13–15], and have indicated that the AO subtype may deteriorate at a slower pace in comparison to infantile or juvenile forms [16–18].

Clinical manifestations amongst the AO group vary but commonly include vertical gaze palsy, cerebellar ataxia, cognitive decline, dysarthria, dysphagia and psychiatric symptoms [1, 5, 7–9, 14, 15, 19, 20]. Of note, 24% (9/37) of an AO cohort exhibited the comorbidity of dysphagia with subsequent mortality due to silent aspiration [5]. Silent aspiration is defined as the movement of a liquid or solid bolus into the trachea, below the vocal cords, without overt clinical signs such as coughing or choking. Additionally, aspiration risk has been documented in 12.4% of patients along with gastrostomy tube placement in 16% of patients with childhood onset NPC1 who were followed longitudinally [4]. This significant morbidity of dysphagia and aspiration should not be overlooked because of the vital roles swallowing plays in nutrition, airway support, oral gratification, and quality of life.

The literature has emphasized the neurodegenerative nature of NPC1 without an approved treatment by the United States Food and Drug Administration (FDA) [1]. Pharmacological interventions are in development and available through expanded access programs; however, only miglustat use has been demonstrated to have a protective effect on swallowing function [21–24]. Although approved for treatment for NPC by most regulatory agencies, miglustat, a glucosylceramide synthase inhibitor, can be prescribed off-label for treatment of NPC1 in the United States [25]. The FDA has endorsed the consideration of swallowing function as a potential outcome measure in rare disease research [26], and some NPC1 therapeutic trials have included swallowing function as an endpoint to determine dysphagia progression [20–23].

Our main objective of this paper is to investigate swallowing function in an AO NPC1 cohort. We characterized the functional status in this cohort utilizing established measures described in previous NPC1 studies. Secondarily, we explored NPC1-specific characteristics and their potential association with swallowing function.

## Methods

### Study cohort

Between 1997 and 2023, a total of 146 individuals with a confirmed diagnosed of NPC1 were enrolled in the National Institutes of Health (NIH) IRB-approved NPC1 natural history studies (NCT00001367 and NCT00344331) after obtaining consent or assent from the individuals or their guardians. The purpose of initiating this study was to identify clinical or biochemical markers that could be used as outcome measures. Comprehensive visits were conducted at intervals of approximately 6-to-12 months assessing medical status, disease progression, and swallowing function. Visits included the following evaluations: history, physical exam, cranial nerve (oral-motor assessments) [27], and videofluoroscopic swallowing studies (VFSS), when clinically indicated.

Of the 146 enrolled individuals, 14 met inclusion criteria for our analysis, which required neurological onset after the age of 15 years and completion of swallowing evaluations. Patients who had received investigational treatments in development or through expanded access (Arimoclomol and Adrabetadex, respectively) were excluded from our analysis due to their unknown effects on swallowing. However, patients receiving miglustat were included due to its reported protective effect on swallowing function [4, 16, 20–24]. All individuals were assessed at baseline, and 6 (42.3%) were followed longitudinally for a total of 24 evaluations. Attempts were made for yearly follow-up in this study, but attrition was experienced with varying lengths of time between visits.

### Outcomes

#### Medical history and physical evaluation

During each study visit, a standardized and NPC1-specific medical history was gathered by conducting interviews with the individual, parent or guardian and reviewing relevant medical records, if available. The baseline visit involved a comprehensive assessment covering various aspects such as phenotypic presentation, diagnostic history, seizure history, and pharmacological interventions. This information helped determine the onset of NPC1-specific symptoms, neurological symptoms, seizures, diagnostic delays, and miglustat use. For our analysis, we categorized peripheral disease symptoms as NPC1-specific symptoms [1, 5, 7–9, 14, 15, 19, 20] and symptoms originating from the central nervous system as neurological symptoms, excluding psychiatric symptoms.

Additionally, the NPC1 neurological severity score (NSS) was used to assess the severity of neurological symptoms. The NSS, based on Yanjanin et al.'s description [28], was scored based on information obtained from the interview conducted at each visit. It encompasses nine major domains (ambulation, cognition, eye movement, fine motor skills, hearing, memory, seizures, speech, and swallowing) and eight minor domains (auditory brainstem response, behavior, gelastic cataplexy, hyperreflexia, incontinence, narcolepsy, psychiatric issues, and respiratory problems). We specifically focused on domains that related to swallowing function, which included ambulation, speech, swallowing, fine motor skills, cognition, memory, seizures, cataplexy, narcolepsy, behavior, psychiatric issues, and respiratory problems. The total NSS was calculated by summing scores from all domains included in the scale.

#### Swallowing evaluations

Since dysarthria and dysphagia have been prominently noted in the AO cohort, a speech-language pathologist (SLP) at the NIH evaluated swallowing function at each participant’s visit. Additionally, individuals/proxies were interviewed by an SLP and completed questionnaires to identify possible triggers of dysphagia and determine if further assessment with a VFSS was necessary. Common triggers for VFSS included reported swallowing difficulties, intermittent coughing or choking, challenges swallowing various textures, and issues with bolus transit. VFSS was systematically conducted using various textures (liquid, puree, and solid), and swallowing function was evaluated with 26 parameters of impairments [11]. Two post-hoc interpretive scales, American Speech-Language-Hearing Association National Outcome Measures Scale (ASHA-NOMS) [29] and the NIH Penetration and Aspiration Scale (NIH-PAS) [30] were utilized to document a individual’s ability to swallow safely and identify aspiration risk. The ASHA-NOMS swallow domain, ASHA-NOMS dietary modification subdomain, and NIH-PAS were included in our analysis to examine AO NPC1 disease progression and note associations with other clinical measures. The scales for ASHA-NOMS, NIH-PAS, solid modifications, and liquid modifications were reversed to align with increasing severity direction and were then analyzed accordingly, similar to Solomon et al. [4].

#### Sample collection and neurofilament light chain assay

In the NIH NPC1 natural history study, all but two AO patients underwent a lumbar puncture during the baseline visit to obtain a cerebral spinal fluid (CSF) sample (*n* = 12). In our analysis, we included the neurofilament light chain (NEFL) and ubiquitin carboxyl-terminal esterase L1 (UCHL1) levels in the CSF of a subgroup of patients (*n* = 9) who were previously measured in Agrawal et al. in 2023 and Cawley et al. in 2023 [31, 32].

### Statistics

Data are described using frequency (percentage) and descriptive statistics [mean ± SD or median (IQR)]. All data were assessed for distributional assumptions and analyzed accordingly. Categorical data between groups (e.g. sex, follow-up) were compared by Fisher’s Exact tests, and if ordinal (e.g., PAS, ASHA-NOMS) using the Cochran-Armitage Trend test. Continuous data were compared between groups by t-tests or Wilcoxon rank sum tests, as appropriate. Correlation analyses utilized Spearman’s rho. Longitudinal data were descriptive. Regression models assessed the relation between NEFL and UCHL1 and NPC1 outcomes with age as a covariate. Statistical evidence was assessed by magnitude of differences, data variability, and *p*-values. Data were analyzed using SAS v9.4 (SAS Institute, Inc, Cary, NC).

## Results

### Baseline characteristics of adult onset participants

Baseline characteristics of AO NPC1 participants are reported in Table 1, demonstrating a predominately female cohort (11/14, 78.6%). Prior to diagnosis with NPC1, the average diagnostic delay was 9.3 ± 8.8 years. At the baseline visit for the NIH natural history study, the mean age was 43.1 ± 16.7 years with the average onset of first NPC1-related symptoms at 30.8 ± 13.8 years. Among all participants, the first NPC1-related symptom was neurologically based with no reported history of hepatosplenomegaly, typically reported in the younger onset cohorts [4]. The average age of onset of first NPC1-related symptoms and neurological symptoms were 30.8 ± 13.8 years. The average duration of neurological symptoms was 12.2 ± 7.9 years at baseline. Two participants (14.3%) had a history of seizures with onset at 18.0 and 2.0 years old. Of these two patients, only one (50%) was taking seizure medications. In terms of other medications, five patients (35.7%) were on miglustat and seven (50%) on psychiatric medications at baseline.

**Table 1:** Characteristics for adult-onset NPC1 patients at baseline

Characteristic	Overall cohort (*n* = 14)	Females (*n* = 11)	Males (*n* = 3)	*P*-value (F vs M)	Baseline data of patients with follow-up (*n* = 6)	*P*-value (With vs Without Follow-up)
**Demographic**						
*Sex, Males, n (%)*	3 (21.4%)	-	-	-	2 (33.3%)	0.54
*Age at baseline visit, mean (SD), y*	43.1 (16.7)	48.7 (13.9)	22.2 (4.6)	**0.008**	33.0 (11.8)	**0.046**
*Patient with follow-up data, n (%)*	6 (42.9%)	4 (36.4%)	2 (66.7%)	0.54	-	-
**Disease**						
*Diagnostic delay, mean (SD), y*	9.3 (8.8)	12.2 (7.3)	-1.3 (4.9)	**0.011**	6.7 (9.0)	0.36
*Age of onset of first NPC-related symptoms, mean (SD), y*	30.8 (13.8)	33.8 (14.0)	19.7 (4.2)	0.12	21.8 (8.7)	**0.033**
*Age of onset of neurological symptoms, mean (SD), y*	30.8 (13.8)	33.8 (14.0)	19.7 (4.2)	0.12	21.8 (8.7)	**0.033**
*Duration of neurological symptoms at baseline, mean (SD), y*	12.2 (7.9)	14.9 (6.7)	2.5 (0.5)	**< 0.001**	11.1 (7.1)	0.67
*Patients with a history of seizures at baseline, n (%)*	2 (14.3%)	2 (18.2%)	0 (0%)	NA	1 (16.7%)	1.0
*Patients taking seizure medications, n (%)*^a^	1 (50%)	1 (50%)	0 (0%)	NA	NA	1.0
*Age of seizure onset, mean (SD), y*^a^	10.0 (11.3)	10.0 (11.3)	NA	NA	NA	1.0
*Duration of follow up (SD), y*	-	2.9 (1.4)	4.1 (3.2)	0.52	3.3 (1.9)	-
*Miglustat use at baseline, n (%)*	5 (35.7%)	4 (36.4%)	1 (33.3%)	1.0	2 (33.3%)	1.0
*Psych medications at baseline, n (%)*	7 (50.0%)	6 (85.7%)	1 (14.3%)	1.0	3 (50.0%)	1.0
**Clinical**						
*ASHA-NOMS, median (IQR)*	0 (0–1)	1 (0–1)	0 (0–0)	0.091	0 (0–1)	0.53
*NIH-PAS, median (IQR)*	0 (0–1)	1 (0–1)	0 (0–0)	0.12	0 (0–1)	1.0
*Silent aspiration from VFSS, n (%)*	1 (7.1%)	1 (9.1%)	0 (0%)	1.0	0 (0%)	0.43
*Total NSS, mean (SD)*	15.6 (6.7)	17.5 (6.4)	9.0 (1.7)	**0.048**	18.7 (8.2)	0.15
*NSS swallow, mean (SD) or median (IQR)*	1.5 (1.3)	2 (1–3)	0 (0–1)	0.066	1.8 (1.5)	0.38
*NSS psych, median (IQR) or median (IQR)*	1 (0–2)	1 (0–2)	0 (0–2)	0.66	1.0 (1.1)	-
*NEFL levels in CSF, mean (SD)*	1051.1 (426.5)	1051.1 (426.5)	NA	NA	828.9 (161.8)	0.18
*UCHL1 levels in CSF, mean (SD)*	1682.2 (490.6)	1682.2 (490.6)	NA	NA	1752.7 (583.4)	0.73

Continuous data were compared between two groups using t-test or Wilcoxon rank sum test. Categorical data were compared by Fisher’s exact test or chi-square tests for trend if ordinal

*ASHA-NOMS* American Speech-Hearing Association National Outcomes Measure Score*, CSF* Cerebral Spinal Fluid*, IQR* Interquartile Range*, NIH-PAS* National Institutes of Health Aspiration and Penetration Score*, NEFL* Neurofilament Light Chain*, NSS* Neurological Severity Scale*, SD* Standard Deviations*, y* Years*, VFSS* Videofluoroscopic Swallow Study*, UCHL1* Ubiquitin Carboxyl-Terminal Esterase L1

^a^These calculations were based on patients who had seizures at baseline (*n* = 2) and at follow-up (*n* = 1)

### Comparison of baseline characteristics by sex

Given the higher number of females in our cohort, we conducted further analysis of baseline clinical characteristics by sex (Table 1). On average, females in our cohort were 2.19 times older than the males (*p* = 0.008), experienced a substantially longer diagnostic delay (*p* = 0.011), and had a longer duration of neurological symptoms at baseline visit (*p* < 0.001). They also appeared to exhibit higher total NSS scores (*p* = 0.048) compared to males. Furthermore, although not significant, females tended to report higher NSS swallow scores (*p* = 0.066), which aligns with increased swallowing complaints. However, there were no sex differences observed across the other baseline characteristics.

### Comparison of baseline characteristics by patients with follow-up

To further characterize the AO cohort, we compared baseline characteristics between patients with follow-up data (6/14, 42.9%) and those without. Those who returned for follow-up tended to be younger at baseline visit (*p* = 0.046) and experienced an earlier onset of NPC1-specific and neurological symptoms (*p* = 0.033). However, no other differences were observed.

### Swallowing outcomes

**Fig. 1:**
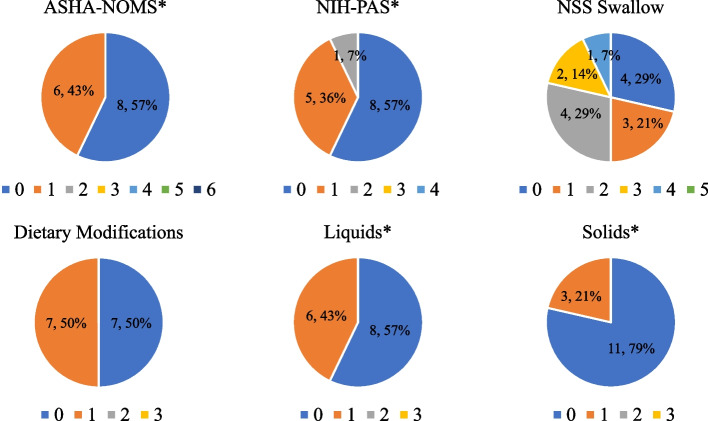
Frequency of swallowing outcome scores of adult-onset cohort at baseline visit. *These swallowing outcomes scales were reversed and transformed to make them consistent with the direction of all rating scales used in the analysis. For all scales utilized, a score of 0 signifies safe independent swallowing function, no aspiration risk, normal (no dysphagia), or no dietary modifications. *ASHA-NOMS, American Speech-Hearing Association National Outcomes Measure Score; NIH-PAS, National Institutes of Health Aspiration and Penetration Score; NSS, Neurological Severity Scale*

The frequency of swallowing outcome scores for the ASHA-NOMS swallow domain, ASHA-NOMS dietary modifications subdomains, and NIH-PAS at baseline for AO patients are depicted in Fig. 1. For all scales utilized to assess swallowing function and dietary modifications, a score of 0 signifies safe independent swallowing function, no aspiration risk, or no dietary modifications. The median (IQR) scores for ASHA-NOMS swallow domain and overall dietary modification subdomains were 0 (0–1) and 1 (0–1), respectively. Within the dietary modification subdomains, the liquid subdomain score was 0 (0–1), and the solid subdomain score was 0 (0–0). Table 2 reveals that there were no correlations between the ASHA-NOMS swallow domain and diagnostic delay (*r*_s_ = 0.22, *p* = 0.43), onset of the first NPC1-specific symptoms (*r*_s_ = -0.05, *p* = 0.86), age of neurological onset (*r*_s_ = -0.05, *p* = 0.86), and duration of neurological symptoms (*r*_s_ = 0.31, *p* = 0.27). Furthermore, we found no associations between the ASHA-NOMS swallow domain and miglustat use (*p* = 0.39), seizure medications (*p* = 0.31), and silent aspiration (*p* = 0.31).

**Table 2:** Correlations of swallowing outcomes at baseline for the adult-onset cohort

Swallowing outcomes	Spearman's correlation	*P*-value
**ASHA-NOMS**		
Diagnostic delay	0.22	0.43
Onset of first NPC1 symptoms	-0.05	0.86
Age of neurological symptoms onset	-0.05	0.86
Duration of neurological symptoms	0.31	0.27
**NIH-PAS**		
Diagnostic delay	0.26	0.37
Onset of first NPC1 symptoms	-0.1	0.72
Age of neurological symptoms onset	-0.1	0.72
Duration of neurological symptoms	0.3	0.28
**NEFL CSF Levels**		
ASHA-NOMS	-0.36	0.29
NIH-PAS	-0.4	0.24
NSS swallow	-0.05	0.75
**UCHL1 CSF Levels**		
ASHA-NOMS	0.5	0.16
NIH-PAS	0.46	0.20
NSS swallow	0.27	0.46

*ASHA-NOMS* American Speech-Hearing Association National Outcomes Measure Score*, CSF* cerebral spinal fluid*, NIH-PAS* National Institutes of Health Aspiration and Penetration Score*, NEFL* Neurofilament Light Chain*, NSS* Neurological Severity Scale*, UCHL1* Ubiquitin Carboxyl-Terminal Esterase L1

As for delineating aspiration risk from VFSS in the AO cohort, the median (IQR) score for NIH-PAS was 0 (0–1) with silent aspiration identified in only one patient (7.1%) (Table 1). Similar to ASHA-NOMS, no correlations or associations were observed with clinical characteristics (Table 2). NIH-PAS was not correlated with diagnostic delay (*r*_s_ = 0.26, *p* = 0.37), onset of first NPC1-symptoms (*r*_s_ = -0.10, *p* = 0.72), age of neurological onset (*r*_s_ = -0.10, *p* = 0.72), or duration of neurological symptoms (*r*_s_ = 0.30, *p* = 0.28). Moreover, no associations between NIH-PAS and miglustat use (*p* = 0.54), seizure medications (*p* = 0.09), and silent aspiration (*p* = 0.09) were identified.

### Niemann-Pick disease type C neurological severity score

**Fig. 2:**
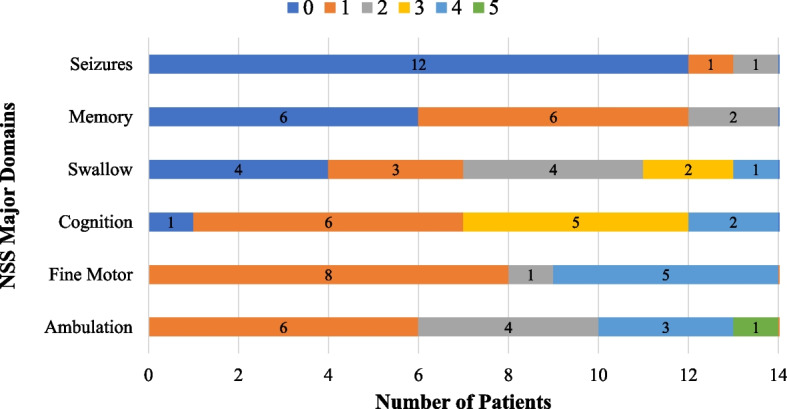
Frequency of NPC1 Neurological Severity Score (NSS) major domain scores for adult-onset NPC1 patients at baseline visit. For NSS major domains, a score of 0 indicates normal functioning, with severity increasing as the score rises. Detailed descriptions of scores for each domain can be found in the NSS Clinical Severity Scale outlined by Yanjanin et al. [28]. *NSS, Neurological Severity Scale*

**Fig. 3:**
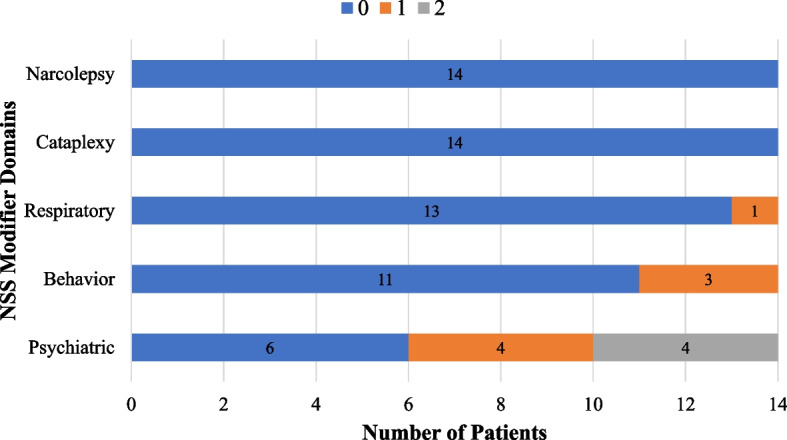
Frequency of NPC1 Neurological Severity Score (NSS) modifier domain scores for adult-onset NPC1 patients at baseline visit. For NSS modifier domains, a score of 0 indicates normal functioning, with severity increasing as the score rises. Detailed descriptions of scores for each domain can be found in the NSS Clinical Severity Scale outlined by Yanjanin et al. [28]. *NSS, Neurological Severity Scale*

For a comprehensive explanation of each score within each domain refer to Yanjanin et al. [28], but a score of 0 across all domains signifies normal or no history. The frequency of NSS major domains for the AO cohort are demonstrated in Fig. 2. The median (IQR) score for the NSS major domains were the following: NSS_Ambulation_ = 2 (1–4), NSS_Speech_ = 2 (2–2), NSS_Swallow_ = 1.5 (0–2), NSS_Fine Motor_ = 1 (1–4), NSS_Cognition_ = 2 (1–3), and NSS_Memory_ = 1 (0–1). As for the NSS modifier domains, the frequencies of them are depicted in Fig. 3. The following were the median (IQR) scores for the minor domains: NSS_Seizure_ = 0 (0–0), NSS_Cataplexy_ = 0 (0–0), NSS_Narcolepsy_ = 0 (0–0), NSS_Behavior_ = 0 (0–0), NSS_Psych_ = 1 (0–2), and NSS_Respiratory_ = 0 (0–0). The mean total NSS score that included all 17 domains was 15.6 ± 6.7 (Table 1). No associations were found between NSS domains and swallowing outcomes, as measured by ASHA-NOMS and NIH-PAS, in the AO cohort, except for a significant association between seizures and NIH-PAS (*p* = 0.026) (Table 3).

**Table 3:** Correlations of NPC1 NSS domains with ASHA-NOMS and NIH-PAS

Swallowing outcome	NSS domain	*P*-value
ASHA-NOMS	Ambulation	0.40
	Speech^a^	0.42
	Swallow	0.50
	Fine Motor	0.20
	Cognition	0.33
	Memory	0.43
	Seizures^a^	0.09
	Behavior	0.74
	Psych	0.61
	Respiratory^a^	0.42
	NSS total	0.24
NIH-PAS	Ambulation	0.31
	Speech^a^	0.43
	Swallow	0.47
	Fine Motor	0.15
	Cognition	0.30
	Memory	0.34
	Seizures^a^	0.026
	Behavior	0.68
	Psych	0.46
	Respiratory^a^	0.43
	NSS total	0.18

*ASHA-NOMS* American Speech-Hearing Association National Outcomes Measure Score*, NIH-PAS* National Institutes of Health Aspiration and Penetration Score*, NSS* Neurological Severity Scale

^a^These domains only had one patient who had a deviation from 0. Cataplexy and narcolepsy were excluded due to no deviation from 0

### Swallowing outcomes correlations with CSF biomarker levels

Of the 9 CSF samples analyzed at baseline, all patients were female (*n* = 9, 100%). The mean (SD) NEFL and UCHL1 levels were 1051.1 ± 426.5 and 1682.1 ± 490.6, respectively. For NEFL, no correlations were observed with ASHA-NOMS (*r*_s_ = -0.24, *p* = 0.51), NIH-PAS (*r*_s_ = -0.29, *p* = 0.43), and NSS swallow domain (*r*_s_ = 12, *p* = 0.75). Results were similar when these associations adjusted for age. Similarly, for UCHL1, there were no correlations with ASHA-NOMS (*r*_s_ = 0.50, *p* = 0.16), NIH-PAS (*r*_s_ = 0.46, *p* = 0.20), and NSS swallow domain (*r*_s_ = 0.27, *p* = 0.47) were denoted.

## Discussion

In this manuscript, we conducted a comprehensive prospective assessment of the swallowing function in the largest group of AO NPC1 patients to date. Our cohort comprised of 14 individuals, predominantly female (*n* = 11, 78.6%), with an average age of 43.1 ± 16.7 years at the initial visit. Upon further examination, we uncovered notable disparities in age at baseline, diagnostic delay, duration of neurological symptoms, and total NSS based on sex. We also identified variations in age at baseline, onset of the first NPC-related symptoms, and age of neurological symptoms between patients with follow-up visits and those without. Overall swallowing abilities remained highly functional with minimal dietary modifications and no overt signs of aspiration or silent aspiration. In this study, in contrast to larger prior studies including younger individuals with NPC1 [5], we found no significant correlations between the swallowing outcomes (ASHA-NOMS, NIH-PAS, NSS swallow domain) and NPC1-associated biomarkers. This could represent the milder presentations seen in the AO cohort.

Our results provide a thorough analysis of swallowing abilities with potential explanations for the key findings. We did not anticipate a sex difference in the enrollment with a higher female to male ratio than previously reported [5, 8]. Despite this uneven distribution, we do not believe that sex differences have any meaningful disease specific relevance based on prior literature on AO NPC1 and other cohorts of NPC1 (early onset and late childhood onset) [1, 4, 8]. Moreover, further studies involving larger cohorts of AO NPC1 patients are required to confirm these observed sex differences. The mean diagnostic delay and duration of neurological disease amongst the AO cohort were 9.3 ± 8.8 years and 12.2 ± 7.9 years, respectively— consistent with the NPC1 literature relative to AO cases [1, 5]. Notably, there is a large SD due to the inclusion of one adult male patient who received an earlier diagnosis compared to the other patients, prompted by his sibling’s diagnosis and subsequent enrollment in the natural history study. This fortuitous inclusion allows for a more comprehensive characterization of adult NPC1 phenotypes. However, the inclusion of this male patient, alongside the small cohort size, may account for the significant differences observed between sexes in terms of diagnostic delay, duration of neurological symptoms, and total NSS. It is important to note that the duration of neurological symptoms and total NSS may have been influenced by heightened awareness of NPC1 symptomatology within the family.

Similar to others who have reported on the AO cohort, we identified our patients to have a mean age of neurological onset of 30.8 ± 13.8 years with variability in diagnostic delay [1, 5, 8]. The wide SD in our mean diagnostic delay, 9.3 ± 8.8 years, may be multifactorial and be explained by the known heterogeneity of NPC1, unique psychiatric and motor initial dysfunction, and lack of medical awareness of the AO NPC1 presentation. These factors often result in misdiagnosis and delayed diagnosis as reported in Sevin et al. [8] and Nadjar et al. [5, 8]. Our findings depict a truly AO cohort as the literature often combine results of adolescent and adults together within their analyses. Further analysis of the disease-specific characteristics within our AO cohort revealed that the age of onset of first NPC1-related symptoms was identical to the age of neurological symptoms onset. This further supports the differences in clinical presentation between AO and other NPC1 subgroups [early-childhood (ECO) and late-childhood (LCO) onset] [4].

In both the ECO and LCO patient groups enrolled in the NIH NPC1 natural history study, we observed a significant attrition of patients during follow-up. This pattern was similarly evident in our AO cohort. Thus, we conducted a comparative analysis of baseline findings between patients who continued with follow-up and those who did not. Interestingly in our cohort, individuals who returned for follow-up were younger at the baseline line visit than those without follow-up, this appeared to be driven by the male cohort. Not surprisingly, those who returned for follow-up exhibited earlier onset of neurological symptoms than those with only a baseline visit. It is likely that patients who were younger at baseline visit may have more parental/caregiver support and flexibility in education or occupational commitments than those who are older, allowing them to travel and complete repeated NPC1 assessments at NIH. We do not believe psychiatric or disease progression contribute to these differences.

Given the recent literature on migulstat and its protective effect on swallowing function in NPC1 ECO and LCO groups, we investigated the use of miglustat within our cohort. We found that 5 patients (35.7%) were taking miglustat at baseline visit, which differed from Nadjar et al.’s AO cohort (26/35, 74.3%) [5]. We attribute this difference to the accessibility of miglustat in the United States, as it is currently not FDA-approved and there are difficulties in obtaining health insurance coverage and reimbursement. However, in Europe, miglustat has been approved for NPC1 with neurological manifestations since 2009. Considering the potential sedative effects of psychiatric medications affecting swallowing function, we noted that 7 patients (50%) were using such medications at the baseline visit, without any significant associations with swallowing dysfunction. We acknowledge the limitations of the data set requiring further longitudinal analysis within the AO cohort.

This manuscript characterizes swallowing abilities in adults with NPC1 to enhance the understanding of potential dysfunction. While swallowing function in NPC1 has been previously reported, limited qualitative and quantitative analysis has been completed in the AO cohort. In this study, we utilized VFSS to identify oropharyngeal impairments that were summarized via interpretive scores (ASHA-NOMS and NIH-PAS) to assist in maintaining quality of life and patient safety. Overall, our AO patients were able to swallow independently with no/minimal cueing with six patients (43%) avoiding specific food items or requiring more time. Upon risk analysis of aspiration, the cohort demonstrated no obvious aspiration risk or laryngeal aspiration in eight patients (57%), minimal risk with intermittent laryngeal penetration and retrograde excursion in five patients (36%), and only one with moderate risk (7%). Dietary modifications were recommended in seven patients (50%), particularly for liquid viscosities (*n* = 6, 43%) rather than solids (*n* = 3, 21%).

Despite variability in the NSS swallow scores, this measure provides a more comprehensive understanding of a patient’s swallowing status beyond the clinical setting. There were no associations found between these swallowing measures and any NPC1-related parameters or the NSS domains at the baseline visit. In contrast to the literature on AO NPC1, we did not observe severe dysphagia in any of our patients, which is associated with mortality due to silent aspiration [5]. While the symptoms of dysphagia are evident, the comparatively milder nature of our cohort when compared to other documented cases may be linked to the acknowledged heterogeneity in NPC1 and a potential rise in medical awareness, leading to earlier evaluations within our group.

Recent research efforts have concentrated on investigating potential disease-associated biomarkers to track NPC1 clinical disease progression and therapeutic response [31, 32]. Although NEFL and UCHL1 have not been previously associated with swallowing function, we were curious to explore its potential association in this specific subgroup. However, our findings, indicating no associations between swallowing outcomes and NEFL and UCHL1 biomarkers, were consistent with previous reports [31, 32]. Similarly, we attribute these lack of associations to the sensitivity of the biomarkers to denote subtle changes in swallowing function with ASHA-NOMS and NIH-PAS as well as the small sample size.

Similar to other NPC1 cohorts, the AO group displayed heterogeneity in both overall swallow safety and the risks of aspiration. We acknowledge the limitation of our study design in the small sample size; however, the importance of assessing swallowing function in this population cannot be understated. Our recommendations should be interpreted with caution keeping in mind the limited sample size. Any type of swallowing dysfunction can significantly affect an individual's lifestyle and overall health, raising concerns for both patients and their families. Furthermore, the literature underscores the considerable morbidity and mortality linked to aspiration pneumonia in the context of swallowing dysfunction in progressive neurological conditions [33]. This highlights the critical importance of assessing and managing one's swallowing ability, placing it at the forefront for clinical consideration in patients with NPC1.

Given our extensive clinical and research backgrounds in NPC1, we advocate that AO patients undergo questioning that investigates potential swallowing concerns, with triage as needed. The following recommendations are offered to both patients and professionals:

- Clinical monitoring with completion of the NSS swallow domain should be performed at all healthcare visits.
- NPC1 patients should undergo an initial swallowing assessment by a SLP upon diagnosis, with subsequent yearly reassessments. VFSS should be considered when clinically warranted due to concerns of dysphagia and its comorbidities previously reported [5].
- Adherence to SLP-recommended therapeutic protective techniques, proper positioning, utensil modifications, and alternative feeding methods is crucial for patients and their families across home and social environments.
- Patients and their families should receive education on swallowing function, precautions against aspiration, and various management strategies.

## Conclusion

Despite the rarity of NPC1, our understanding of swallowing dysfunction in this disease is steadily advancing. This manuscript on AO swallowing characterization establishes the groundwork for understanding the heterogeneous presentation of swallowing ability, specifically in terms of overall safety in the ability to eat and aspiration risk. Unlike the phenotypic expression in the ECO and LCO cohorts, we were unable to identify any associations between the swallowing outcomes (ASHA-NOMS, NIH-PAS, NSS swallow) and NPC1-related parameters and known biomarkers. Although our findings are limited and require further longitudinal analysis within this cohort, this manuscript suggests the need for comprehensive swallowing assessments and management to reduce the known morbidities associated with AO patients, such as aspiration and silent aspiration.

## Data Availability

The data that support the findings of this study are not openly available due to reasons of sensitivity and are available from the corresponding author upon reasonable request. Data are located in controlled access data storage at NIH.

## Abbreviations

ASHA-NOMS: American Speech Hearing Association National Outcomes Measures Scale
AO: Adult onset
CSF: Cerebral spinal fluid
FDA: Food and Drug Administration
ECO: Early-childhood Onset
IRB: Institutional Review Board
IQR: Interquartile ranges
LCO: Late-childhood Onset
NEFL: Neurofilament light chain
NIH: National Institutes of Health
NIH-PAS: National Institutes of Health Penetration and Aspiration Scale
NPC1: Niemann-Pick Type C1
NSS: Neurological Severity Score
SD: Standard deviation
SLP: Speech language pathologist
UCHL1: Ubiquitin carboxyl-terminal esterase L1
VFSS: Videofluoroscopic Swallow Study
